# Assembly and Folding Properties of Cytosolic IgG Intrabodies

**DOI:** 10.1038/s41598-020-58798-7

**Published:** 2020-02-07

**Authors:** Youngsil Seo, Yeonjin Lee, Minjae Kim, Hyunjoon Park, Myung-Hee Kwon

**Affiliations:** 1 Department of Biomedical Sciences, Graduate School, Ajou University, 206 World cup-ro, Yeongtong-gu, Suwon, 16499 Gyeonggi-do South Korea; 2 Department of Microbiology, Ajou University School of Medicine, 206 World cup-ro, Yeongtong-gu, Suwon, 16499 Gyeonggi-do South Korea

**Keywords:** Protein folding, Protein folding, Protein folding

## Abstract

Intrabodies, antibodies expressed within cells, offer an interesting way to target intracellular molecules, making them potentially useful for biotechnology and medicine. However, it remains controversial whether full-size IgG intrabodies expressed in the reducing environment of the cytosol of mammalian cells are workable and structurally sound. Herein, we settle this issue with a systematic investigation of the structure and functionality of four chimeric IgG1s with distinct variable (V) domains but identical constant (C) domains. Full-size IgGs expressed in the cytosol of HEK293 cells were either assembly-competent or -incompetent, depending on the intrinsic properties of the V regions. Structural integrity of the C region is required for H:L association and the formation of a functional antigen-binding site. Partial intrachain disulfide bond formation occurs in both H and L chains of cytosolic IgG intrabodies, whereas interchain disulfide bond formation was absent and dispensable for functional assembly. IgG1s expressed in the cytosol and via the ER were shown to assemble differently. Our findings provide insight into the features and possible utilization of full-size IgGs as cytosolic antibodies in biotechnological and medical applications.

## Introduction

The cytoplasm and nucleus disfavor the oxidation of cysteine thiols in proteins because the thiol-disulfide redox potential is too low to drive formation of stable disulfide bonds, and there is a deficiency in enzymes that catalyze protein thiol oxidation in these cellular compartments^1–4^. Therefore, the formation of stable disulfide bonds in proteins in the cytoplasm and nucleus is extremely rare and, in general, cytoplasmic proteins do not contain disulfide bonds^5^. Although transient formation of disulfide bonds is found in a few redox-sensitive cytoplasmic proteins such as oxidoreductases and transcription factors, these transient disulfide bonds are related to the control of various metabolic and signaling pathways upon sensing oxidants under oxidative stress conditions^6,7^. This transient and reversible oxidant-mediated disulfide bond formation in the cytosol does not contribute to the stability of the native state of proteins^2,3,7^. By contrast, stable and structural disulfide bonds are common in secreted proteins and the extracellular domains of plasma membrane proteins following their introduction by thiol-disulfide oxidoreductases^8^ that act only in oxidative intracellular compartments such as the endoplasmic reticulum (ER)^1,4,9^. Structural disulfide bonds are essential for maintaining the native structure, stability, and/or activity of many proteins, including antibodies^10,11^.

Intracellular antibodies (intrabodies) are antibodies expressed within cells that modulate the functions of antigens by interacting with them inside cells. The use of intrabodies to target intracellular molecules in living cells is a valuable application in biotechnology, medicine, and cell biology^12^. Most intrabodies employed to date are monovalent recombinant single chain variable fragment (scFv) antibodies composed of a variable heavy chain (V_H_) domain and a variable light chain (V_L_) domain connected by a peptide linker. This simple structure has only two intrachain disulfide bonds. However, expression of intrabodies that function in the reducing environment of the cytoplasm and nucleus remains a major challenge, and special strategies are needed to obtain scFvs that are properly folded and functional without disulfide bonds^13–16^. Even more severe usability issues arise for in-cell workable full-size antibodies such as human IgG1 that has 16 (four interchain and 12 intrachain) disulfide bonds. To date, a few cases of the functional assembly of intrabodies expressed in the cytosol of mammalian cells have been reported for full-size IgMs and IgGs^17–19^. However, these studies mainly focused on the targeting of cytosolic molecules by intrabodies; they did not determine whether targeting could be generalized to other full-size cytosolic antibodies, or explore the molecular features of these antibodies. Thus, it would be useful to better understand the functional and structural properties of full-size IgG intrabodies to extend their use as targeting agents for cytosolic molecules.

In the present study, we aimed to investigate the functional and structural features of IgGs expressed in the cytosol of HEK293 cells using four chimeric IgG1 antibodies with distinct V_H_ and V_L_ mouse antibody domains and identical C regions (Cγ1 and Cκ) from human IgG1. Three chimeric IgG1 antibodies (2C281, 6C407, and 10C358) are specific for kinesin family member C1 (KIFC1), and one chimeric IgG1 (3D8) is specific for nucleic acids. Differences in the ability of the four chimeric IgGs to assemble into functional molecules were investigated, along with differences in the oxidation-reduction (redox) state of H and L chains, the effect of the C domain on functional assembly, and the assembly properties of cytosol-directed and ER-directed IgGs. The findings expand our understanding of functional and structural aspects of full-size IgG intrabodies, and their potential uses.

## Results

### V region sequences influence H:L association and antigen-binding in IgGs expressed in the cytosol

**Figure 1:**
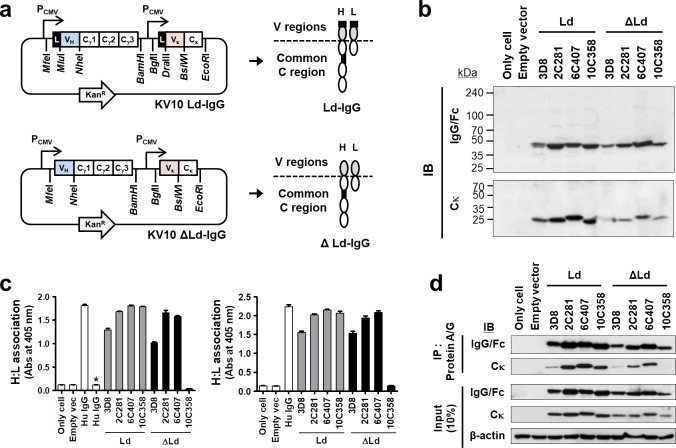
H:L association and antigen-binding of cytosolic IgG1s are determined by the V regions. (**a**) Plasmid constructs for expression of chimeric IgGs. (**b**) Immunoblotting for detection of H and L chains in lysates of HEK293 cells. (**c**) Evaluation of H:L chain association by ELISA using anti-human IgG/Fc (left panel) or anti-human Cκ (right panel) as a capture antibody. Asterisks (*) indicate negative controls included to ensure that the anti-rabbit IgG/Fc-specific antibody does not directly react with human IgG/Fc. Data are presented as mean ± SEM, n = 3. (**d**) Co-IP of H and L chains. Lysates of transfectants were immunoprecipitated using Protein A/G-agarose. Input and IP samples were resolved by reducing SDS-PAGE and probed with the indicated antibodies. Input represents 10% of the total amount of lysate used in for IP. IP, immunoprecipitation; IB, immunoblotting.

Using a single-vector strategy for simultaneous expression of H and L chains (Fig. 1a), four chimeric IgG1s comprising one IgG specific for nucleic acid (3D8) and three IgG1s specific for KIFC1 (2C281, 6C407, and 10C358) were expressed in HEK293 cells in the presence (Ld) and absence (ΔLd) of a leader sequence. In chimeric IgG1s, the V regions are distinct and the C regions (Cγ and Cκ) are identical. Expression of H and L chains of IgG1s in the reducing and denaturing conditions of cell lysates was confirmed by immunoblotting (Fig. 1b).

To assess the association between H and L chains (H:L association) of IgG1s, we prepared lysates of HEK293 cells transfected with plasmids encoding IgGs and subjected them to sandwich enzyme-linked immunosorbent assay (ELISA) and immunoprecipitation (IP). The same lysates used for Fig. 1b were subjected to sandwich ELISA in which wells were coated with anti-human IgG/Fc (Fig. 1c, left panel) and anti-human Cκ (Fig. 1c, right panel) as a capture antibody. Both types of sandwich ELISA revealed H:L association in lysates of ΔLd-3D8, ΔLd-2C281 and ΔLd-6C407, but not ΔLd-10C358 (Fig. 1c). To confirm this H:L association pattern, we transfected HEK293 cells with plasmids encoding IgGs and performed IP with Protein A/G. Precipitated samples in reducing and denaturing conditions were analyzed by immunoblotting. The H chains of ΔLd-3D8, ΔLd-2C281, and ΔLd-6C407 were able to pull down their kappa L chains from cell lysates (Fig. 1d), indicating the successful association of H and L chains. By contrast, the H chain of kappa L chains ΔLd-10C358 was unable to pull down its L chain, indicating a failure to associate. These results demonstrate that the H:L association of IgG1s expressed in the reducing cytosol of mammalian cells is determined by the intrinsic sequence (or properties) of the V regions.

**Figure 2:**
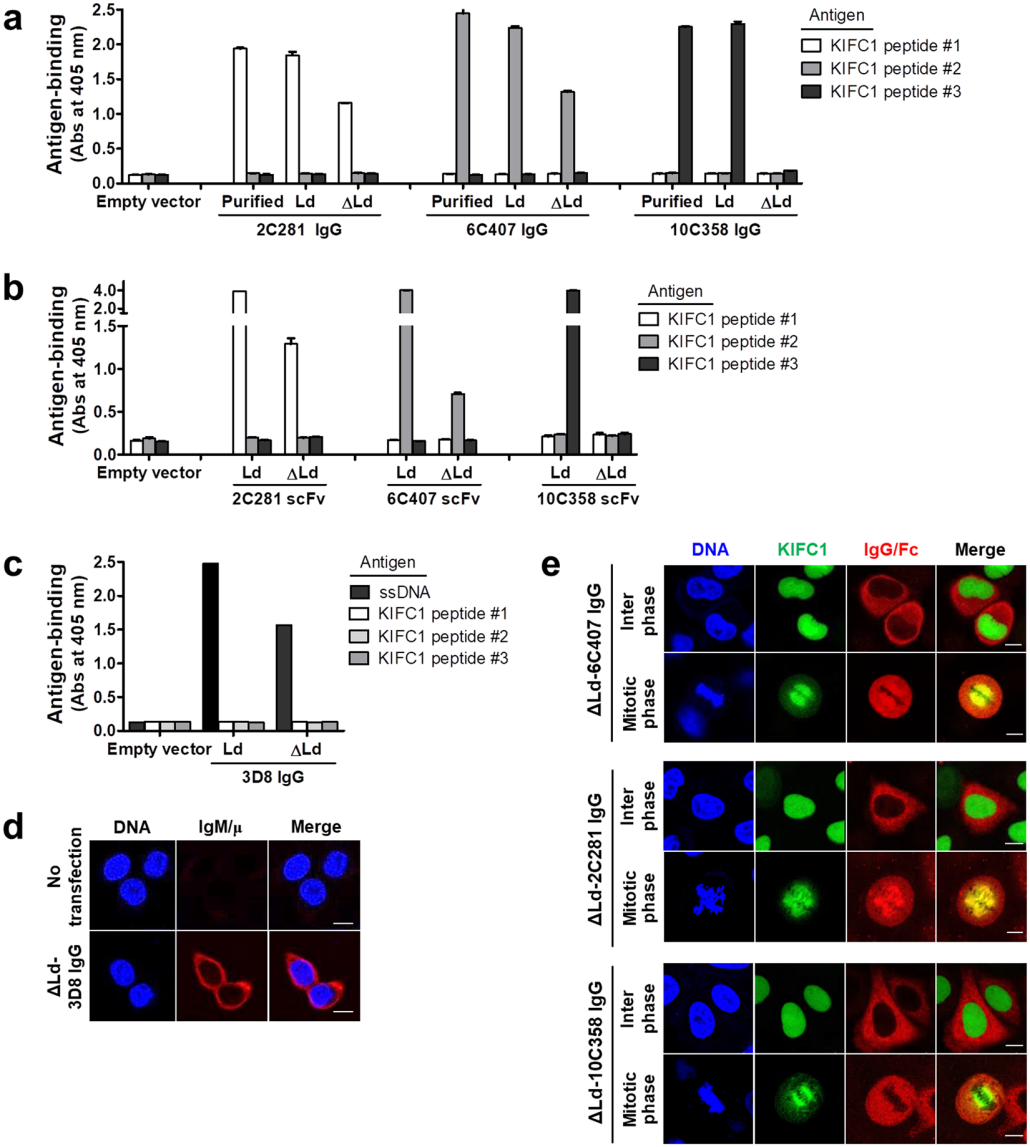
Antigen-binding analyses of IgG1s expressed in the cytosol. (**a**–**c**) Evaluation of antigen-binding activity by ELISA. Lysates of transfectants were placed in wells coated with specific antigens, and bound IgGs were detected with AP-conjugated anti-human IgG/Fc. Bound scFvs labeled with HA tag were detected with anti-HA tag followed by AP conjugated anti-rabbit IgG/Fc. Data are presented as mean ± SEM, n = 3. (**d**) Confocal microscopy analysis of antigen-binding site formation in 3D8 IgG. Transiently transfected HEK293 cells were fixed, permeabilized, and then incubated with O2F3 (mouse IgM), followed by an Alexa Fluor 647-conjugated anti-mouse IgM/μ chain antibody. (**e**) Confocal microscopy analysis of the cellular antigen-binding activity of anti-KIFC1 IgGs. HeLa cells stably expressing GFP-KIFC1 were transfected with the specified plasmids. After synchronization of cells to mitotic phase, cells were fixed and stained with a primary antibody for anti-human IgG/Fc, followed by rhodamine-conjugated anti-goat IgG. Bar = 10 μm.

Next, we assessed the antigen-binding ability of ΔLd-IgG1s by ELISA. The three IgG1s (3D8, 2C281 and 6C407) were able to bind to their specific antigen, and were designated as cytosolic assembly-competent IgG1s. By contrast, ΔLd-10C358, which was incapable of H:L association, was unable to bind to its KIFC1 peptide #3 antigen (Fig. 2a,c), and was designated a cytosolic assembly-incompetent IgG1. Antigen-binding ability of anti-KIFC1 scFv antibodies that are composed of only V regions was similar to that of IgG1s (Fig. 2b), supporting the fact that the antigen-binding ability of cytosolic IgG is determined by the V region. To further confirm that, unlike 10C358, the other three IgG1s (3D8, 2C281, and 6C407) are cytosolic assembly-competent antibodies, we carried out *in situ* immunofluorescence analysis. For analysis of 3D8 IgG, which is an anti-DNA antibody, HEK293 cells transfected with the ΔLd-3D8 IgG1 vector were fixed, permeabilized, and reacted with O2F3 anti-idiotypic antibody that recognizes a conformational epitope of the antigen-binding site of the 3D8 antibody^20^. Fluorescence staining was observed as a diffuse pattern throughout the cytosol, with minimal fluorescence in the nucleus, as expected for a protein localized to the cytoplasm (Fig. 2d). For analysis of 2C281, 6C407, and 10C358 IgG1s that recognize KIFC1, IgGs were expressed in the cytosol of HeLa cells stably expressing GFP-KIFC1, and reacted with anti-IgG/Fc antibody. We observed the cells in mitotic phase because cytosolic IgGs cannot encounter KIFC1 that is localized mainly in the nucleus until the nuclear envelope disappears at the mitotic phase of the cell cycle^21^. Colocalization between KIFC1 and IgG was observed with 2C281 and 6C407, but not with 10C358 (Fig. 2e). As expected, cells in interphase, in which the nucleus and cytosol are separated by the nuclear envelope, did not reveal colocalization between IgG and KIFC1. These results further indicate that 3D8, 2C281, and 6C407 are indeed cytosolic assembly-competent IgG1s, unlike 10C358.

### H:L association of cytosolically expressed IgG1 can occur without correct protein folding

**Figure 3:**
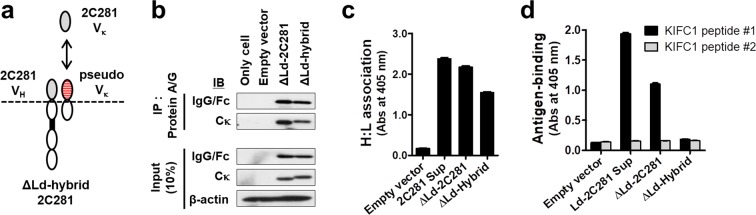
H:L association can occur without formation of the correct antigen-binding site. (**a**) Schematic representation of ΔLd-hybrid IgG1 possessing 2C281V_H_ and pseudo Vκ as V regions. (**b**) Co-IP. Lysates of transfectants were immunoprecipitated using Protein A/G-agarose. Input and IP samples were resolved by reducing SDS-PAGE. Input represents 10% of the total amount of lysate used for IP. β-actin was used as an internal loading control. IP, immunoprecipitation; IB, immunoblotting. (**c**) Evaluation of H:L chain association by ELISA. Lysates of HEK293 transfectants were placed in wells coated with anti-human Cκ, and bound hybrid IgGs were detected with AP-conjugated anti-human IgG/Fc antibody. (**d**) Evaluation of antigen-binding activity by ELISA. Lysates of HEK293 transfectants were placed in wells coated with KIFC1 #1 peptide, and bound chimeric IgGs were detected with AP-conjugated anti-human IgG/Fc antibody. In (**c**,**d**), data are presented as mean ± SEM, n = 6. Sup, culture supernatant.

Failure of ΔLd-10C358 in both H:L association and formation of the correct antigen-binding site prompted us to investigate the correlation between these phenomena. We prepared a lysate of a ΔLd-hybrid 2C281 IgG1 composed of 2C281 V_H_ and an irrelevant pseudo V_K_ region (Fig. 3a), and analyzed H:L association and antigen-binding abilities. The pseudo Vκ gene was obtained from mouse myeloma cell line SP2/0 that is commonly used as a fusion partner for the hybridoma production. Interestingly, IP analysis of the ΔLd-hybrid cell lysate showed that the 2C281 H chain was able to pull down the pseudo kappa L chain, indicating H:L association (Fig. 3b). Furthermore, H:L association in the ΔLd-hybrid lysate was also detected in sandwich ELISA experiments using anti-human Cκ as a capture antibody (Fig. 3c). However, ΔLd-hybrid IgG1 did not bind KIFC1 peptide #1, the specific antigen of 2C281, in ELISA (Fig. 3d). These results demonstrate that H:L association of cytosolic IgG1s can occur irrespective of whether the correct antigen-binding site is formed. Thus, H:L association does not always guarantee that IgGs are correctly folded. These findings also provide further evidence that the intrinsic properties of V regions are the key factor determining H:L association and the formation of the correct antigen-binding site of cytosolic IgG1.

### The structural integrity of the C region affects H:L association and formation of the correct antigen-binding site in cytosolic IgGs

**Figure 4:**
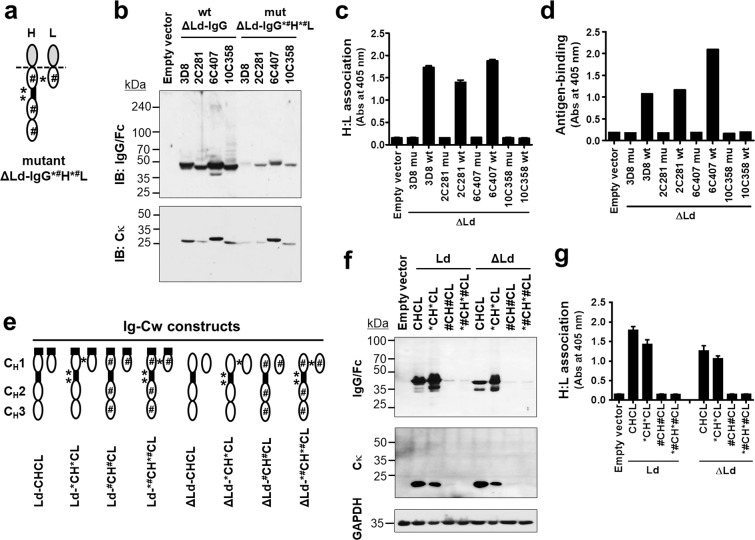
The structural integrity of the C region affects H:L association and antigen-binding of cytosolic IgGs. (**a**) Schematic representation of ΔLd-IgG*^#^H*^#^L. (**b**) Immunoblotting of H and L chains in lysates of HEK293 cells transiently transfected with KV10 vectors encoding WT ΔLd-IgGs and mutant ΔLd-IgG*^#^H*^#^L. (**c**) Evaluation of H:L chain association by ELISA using anti-human Cκ as a capture antibody. (**d**) Evaluation of antigen-binding by ELISA. Lysates of HEK293 transfectants were placed in wells coated with their specific antigens, and bound IgGs were detected with AP-conjugated anti-human IgG/Fc. (**e**) Schematic representation of ΔLd-Ig-Cw mutants. (**f**) Immunoblotting of C_H_ and C_L_ chains. Lysates of HEK293 transfectants were resolved by reducing SDS-PAGE and probed with antibodies recognizing human IgG/Fc or human Cκ light chain. The expression of glyceraldehyde-3-phosphate dehydrogenase (GAPDH) was used as an internal control. (**g**) Evaluation of H:L chain association by ELISA using anti-human Cκ as a capture antibody. In (**c**,**d**,**g**), data are presented as mean ± SEM, n = 3.

We examined the possible involvement of the C region structure in H:L association and formation of the correct antigen-binding site in IgG1s expressed in the cytosol because we thought that distortion of the C region might affect the integrity of the V regions, although the properties of V regions were shown to determine the functional assembly of IgGs when whole C regions (Cw) of H and L chains are intact. We constructed ΔLd-IgG*^#^H*^#^L mutants composed of wild-type (WT) V regions and impaired C regions. In the ΔLd-IgG*^#^H*^#^L mutants, all Cys residues of C regions responsible for inter- and intrachain disulfide bond formation were replaced with Ser residues that are incapable of forming disulfide bonds, and likely cause structural changes. Disruption of inter- or intrachain disulfide bonds is indicated by a superscript asterisk (*) or sharp (#) symbol, respectively, in front of the chain (Fig. 4a). Expression of H and L chains in ΔLd-IgG*^#^H*^#^L mutants was confirmed in reducing and denaturing conditions (Fig. 4b). The same lysates used for Fig. 4b were subjected to ELISA, and the results showed the failure of both H:L association (Fig. 4c) and antigen-binding (Fig. 4d) for all three ΔLd-IgG*^#^H*^#^L mutants of the cytosolic assembly-competent 3D8, 2C281 and 6C407. These results demonstrate that the structural integrity of C regions is required for H:L association and formation of the correct antigen-binding site in ΔLd-IgG1.

To investigate the ability of the C regions themselves in C_H_:C_L_ association in the absence of V regions, we generated truncated IgGs composed only of Cw regions (C_H_1, C_H_2, C_H_3 and C_L_) without a V region, designated as Ig-whole C region (Ig-Cw), in both WT and Cys-to-Ser mutant forms (Fig. 4e). SDS-PAGE under reducing and denaturing conditions followed by immunoblotting revealed a significantly lower level of expression for ^#^C_H_^#^C_L_ and *^#^C_H_*^#^C_L_ compared with C_H_C_L_ and *C_H_*C_L_ (Fig. 4f), indicating that structural impairment caused by disruption of intrachain disulfide bonds in Ig-Cw affects expression level, irrespective of the presence or absence of a leader sequence. The observation of bands smaller than 37.5 kDa for the C_H_ chain of Ld-C_H_C_L_ and ΔLd-C_H_C_L_ may be due to partial degradation. The same lysates used for Fig. 4f were subjected to ELISA, and the results revealed successful C_H_:C_L_ association in both WT C_H_C_L_ and *C_H_*C_L_, whereas the association signal for the Ig-Cw mutants ^#^C_H_^#^C_L_ and *^#^C_H_*^#^C_L_ was not detected (Fig. 4g). The reason we could not detect C_H_:C_L_ association in mutants ^#^C_H_^#^C_L_ and *^#^C_H_*^#^C_L_ may be resulted from low expression level of these mutants and (or) failure in C_H_:C_L_ association. Conclusively, C_H_ and C_L_ chains can associate in the absence of V region and intrachain disulfide bonds in the C region would be important for maintaining structural integrity of C regions.

### Interchain disulfide bonds are absent in H and L chains, and are dispensable for H:L association and antigen-binding site formation in cytosolic assembly-competent IgGs

**Figure 5:**
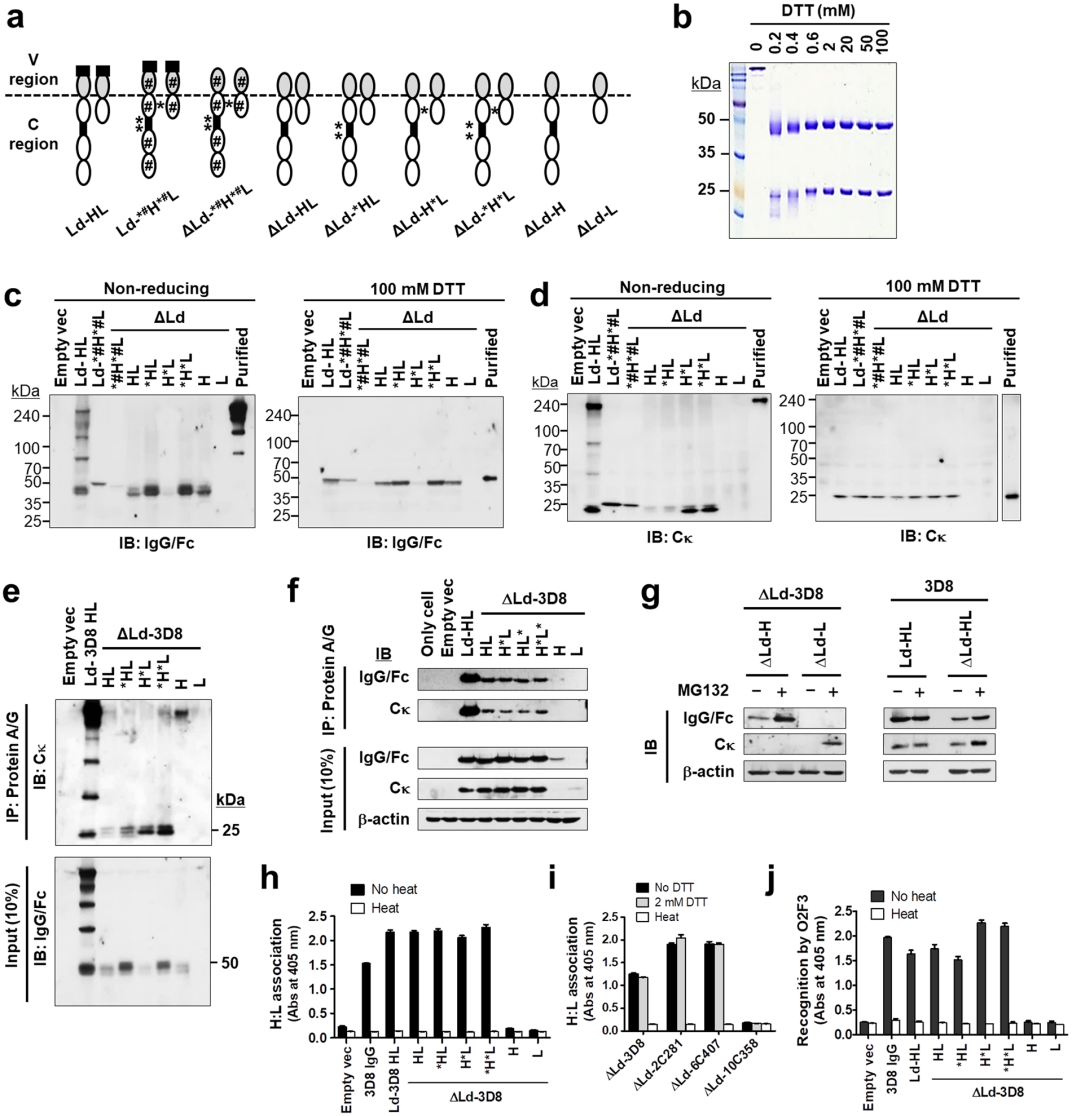
Interchain disulfide bonds are absent and intrachain disulfide bonds are partially formed in cytosolic IgGs. (**a**) Schematic representation of 3D8 IgG1 variants. The absence of interchain and intrachain disulfide bonds is denoted by a superscript asterisk (*) or sharp (#) symbol, respectively, in front of the chain. The locations of asterisk and sharp symbols indicate Cys-to-Ser substitution sites. (**b**) Purified WT 3D8 IgG1 protein was subjected to 15% SDS-PAGE in reducing conditions in the presence of different concentrations of DTT. (**c**,**d**) Immunoblotting. Lysates of HEK293 transfectants were pretreated with 100 mM DTT for 30 min at room temperature (or not), then subjected to non-reducing SDS-PAGE. H (**c**) and L (**d**) chains were detected by immunoblotting with the indicated antibodies. (**e**,**f**) Co-IP of H and L chains. Lysates of transfectants were immunoprecipitated using Protein A/G-agarose. Input and IP samples were resolved by non-reducing SDS-PAGE (**e**) and reducing SDS-PAGE. (**f**) Input represents 10% of the total amount of lysate used for IP. **(g)** Immunoblotting. At 24 hours after transfection, cells were incubated in the presence or absence of 10 µM MG132 for 12 hours. Lysates of transfectants were then resolved by reducing SDS-PAGE. Membranes were probed with antibodies recognizing human IgG/Fc or human Cκ. (**h**,**i**) Evaluation of non-covalent H:L chain association by ELISA. Lysates of transfectants were heated at 100 °C for 10 min or mixed with DTT (final concentration, 2 mM), and then placed in wells coated with Protein L. Captured IgGs were detected with AP-conjugated anti-human IgG/Fc antibody. (**j**) Evaluation of formation of the correct antigen-binding site by ELISA. Lysates of transfectants were placed in wells coated with O2F3 IgM, and bound IgGs were detected with AP-conjugated anti-human IgG/Fc. In (**h**–**j**), data are presented as mean ± SEM, n = 3.

To examine whether inter- and intrachain disulfide bonds are formed in H and L chains of cytosolically expressed IgG1, we generated 3D8 IgG1 variants in which Cys residues involved in inter- and/or intrachain disulfide bond formation were substituted with Ser residues. The inter- and intrachain disulfide bond variants are represented by superscript asterisk (*) and sharp (#) symbols, respectively, in front of the chain (Fig. 5a). Thus, ΔLd-*HL and ΔLd-H*L denote cytosolic 3D8 IgG1 variants that are unable to form disulfide bonds between the two H chains (H-H) or between H and L chains (H-L), respectively, and ΔLd-*^#^H*^#^L denotes the disulfide bond-free mutant in which all Cys residues responsible for the formation of inter- and intrachain disulfide bonds (in V_H_, C_H_1, C_H_2, C_H_3, V_L_ and C_L_) were replaced with Ser residues.

First, we observed changes in protein band patterns when H and L chains were reduced by treating purified 3D8 IgG1 proteins with different concentrations of DTT and subjecting to non-reducing SDS-PAGE. Whole IgG1 antibodies in the absence of DTT formed 200 kDa assemblies, whereas dissociation of H and L chains and/or multiple smeared protein bands corresponding to H and L chains were observed in the presence of 0.2 mM DTT. With 0.2−0.4 mM DTT, H and L chains ran as multiple bands, indicating partially reduced forms, whereas fully reduced H and L chains appeared as a single band at higher DTT concentrations. Oxidized forms of H and L chains migrated faster than their corresponding reduced forms (Fig. 5b).

In transfectant lysates subjected to non-reducing and denaturing conditions, immunoblotting using anti-human IgG/Fc revealed multiple bands, indicating oxidation of H chains to varying extent, with molecular weight (MW) differing by only a few Da around 50 kDa (Fig. 5c, left panel). Likewise, immunoblotting using anti-human Cκ showed two protein bands, suggesting L chains were oxidized to varying degrees, differing in MW by only a few Da near 25 kDa (Fig. 5d, left panel). When lysates of transfectants were subjected to reducing and denaturing conditions (100 mM DTT), H and L chains appeared as a single band pattern, indicating the complete reduction of the chains (right panels of Fig. 5c,d). In the ER-directed Ld-HL, covalently assembled intermediates as well as oxidized free H and L chains were detected. As expected, Ld-*^#^H*^#^L and ΔLd-*^#^H*^#^L showed a single protein band at 50 kDa with anti-IgG/Fc and 25 kDa with anti-human Cκ, irrespective of DTT treatment (Fig. 5c,d). These results suggest that the redox state of H and L chains is a major source of heterogeneity in IgGs, and H and L chains can be partially oxidized in the cytosol. None of the interchain disulfide-bridged IgG forms, such as 2H + 2L, 2H + 1L, 1H + 1L or 2H chains, which would run at a MW much greater than 50 kDa, were detected in any of the ΔLd-transfectants, demonstrating the absence of interchain disulfide bonds in cytosolically expressed IgG1.

To examine interactions between H and L chains expressed in the cytoplasm, we performed IP of transfectant lysates with Protein A/G. Immunoblotting of precipitated material after SDS-PAGE under non-reducing/denaturing conditions showed that partially oxidized H chains were able to pull down partially oxidized L chains from all ΔLd-3D8 transfectant lysates except ΔLd-H and ΔLd-L transfectants expressing H or L alone (Fig. 5e). Non-covalent H:L association of 3D8 IgG1 and its variants were confirmed by IP and immunoblotting in reducing (2 mM DTT) conditions (Fig. 5f). H and L chains appeared as a single band at 2 mM DTT, indicating the complete reduction of Cys residues. These results indicate that non-covalent H:L association of cytosolic assembly-competent 3D8 IgG1 occurs irrespective of the formation of inter- and intrachain disulfide bonds in cytosolically expressed IgG1. Based on input analysis, only traces of H or L chains were detected in lysates of ΔLd-H and ΔLd-L transfectants. Treatment of transfectants with proteasome inhibitor MG132 resulted in a clear increase in H or L chains in these cells. H or L chains expressed in the cytoplasm may be structurally unstable if not associated with each other, and eventually degraded by the proteasome pathway (Fig. 5g).

Non-covalent H:L association in the assembly of competent IgG1s was also verified by sandwich ELISA using a specific Vκ family-binding protein, Protein L, as a capture protein. H:L association was observed in lysates of ΔLd-3D8 and all its variants (Fig. 5h), as well as for other ΔLd-antibodies (2C281 and 6C407) treated with 2 mM DTT (Fig. 5i), but not when they were denatured by heat (negative controls), indicating that non-covalent H:L association of the assembly of competent IgG1s occurs in the cytosol. Moreover, ELISA was performed to investigate the formation of antigen-binding sites in 3D8 IgG variants using the O2F3 anti-idiotypic antibody, and this showed that all 3D8 IgG1 variants were recognized by O2F3, except for variants expressing H or L alone, unless inactivated by heat (Fig. 5j). These results suggest that interchain disulfide bonds are dispensable for the formation of the correct antigen-binding site in cytosolic assembly-competent IgG1.

### Intrachain disulfide bonds are partially formed in H and L chains of cytosolic assembly-competent IgGs

**Figure 6:**
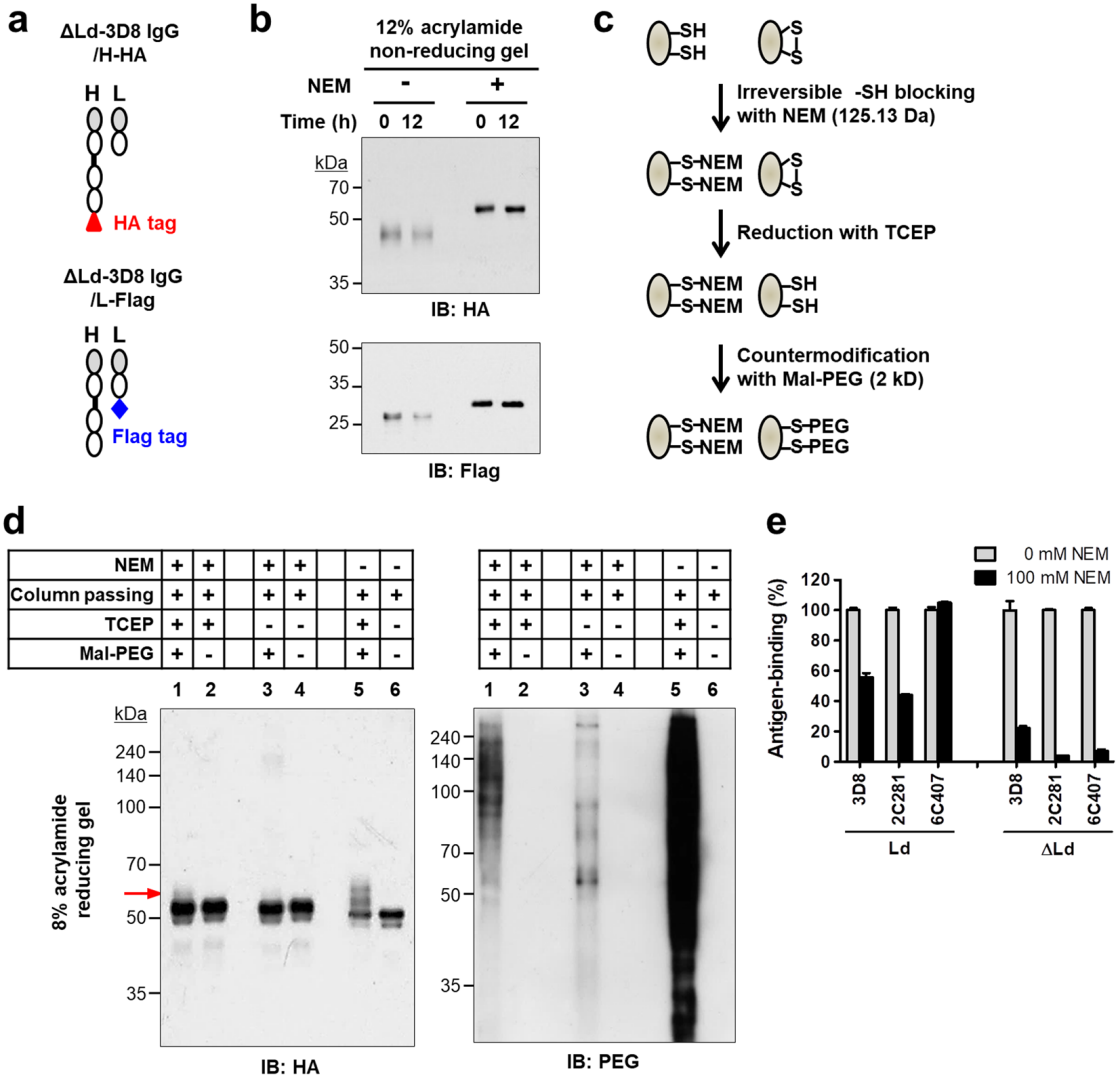
Redox state of H and L chains of IgGs expressed in the cytosol of HEK293 cells. (**a**) Schematic representation of ΔLd-3D8 IgG/H-HA and ΔLd-3D8 IgG/L-Flag proteins. (**b**) Immunoblotting. Lysates of cells transfected with plasmids encoding ΔLd-3D8 IgG/H-HA or ΔLd-3D8 IgG/L-Flag were prepared in the presence and absence of 100 mM NEM and separated by non-reducing 8% or 12% SDS-PAGE, both immediately and after 12 hours incubation at room temperature. H and L chains were detected by immunoblotting with the indicated antibodies. (**c**) Workflow of the assay. (**d**) Immunoblotting. Cells transfected with a plasmid encoding ΔLd-3D8 IgG/H-HA were pre-incubated with NEM at a final concentration of 100 mM at 37 °C for 15 min. Lysates of cells were then prepared in the presence of 100 mM NEM. Lysates were reacted in order with 5 mM TCEP and 10 mM Mal-PEG and separated by reducing SDS-PAGE, and H chain and PEG were detected by immunoblotting with anti-HA and anti-PEG antibodies, respectively. Control samples received different treatments as indicated by + and −. (**e**) Evaluation of antigen-binding activity by ELISA. Lysates of HEK293 transfectants were prepared in the presence of 100 mM NEM, placed in wells coated with specific antigens, and bound IgGs were detected with AP-conjugated anti-human IgG/Fc. Data are presented as mean ± SEM (n = 6).

Although we found that intrachain disulfide bonds may be intrinsically formed in the cytosol, as shown in Fig. 5c,d, the possible formation of spontaneous and non-enzymatic intrachain disulfide bonds upon exposure to air during cell lysate preparation^22^ cannot be ruled out. In order to demonstrate that this was unlikely, we compared the band patterns of H and L chains from freshly prepared and 12 hours air-exposed HEK293 cell lysates made using ΔLd-3D8 IgG vectors expressing HA-tagged H chain and Flag-tagged L chain, respectively (Fig. 6a). A set of transfectant lysates were prepared in the presence of NEM, a membrane-permeable, sulfhydryl-alkylating agent with a MW of 125.13 Da, to block permanently free thiols of Cys residues and prevent disulfide bond formation. Proteins were subjected to immunoblotting analysis, immediately or after exposure to air for 12 hours in reducing and denaturing conditions, using anti-HA and anti-Flag antibodies. No differences were observed in the band patterns for H or L chains in cells not treated with NEM that were exposed to air for 0 or 12 hours. The same was also true for H and L chains in lysates of NEM-treated cells irrespective of the duration of exposure to air, although the mobility was slightly slower than in NEM-untreated lysates due to alkylation (Fig. 6b). These results demonstrate that spontaneous *ex vivo* non-enzymatic disulfide bonds were not formed by molecular oxygen under aerobic conditions in our experimental conditions, in which all assaying of lysates was performed within 12 hours following cell lysis. Therefore, we could exclude the possibility of experimental error by accidental *ex vivo* oxidation of H and L chains during sample preparation. The findings also support the partial formation of intrinsic intrachain disulfide bonds in the cytosol.

Next, to prove disulfide bond formation in the H chain of cytosolically expressed IgGs, we employed a straightforward method to detect the redox state of the protein (Fig. 6c). We transfected HEK293 cells with the ΔLd-3D8 IgG vector that expresses HA-tagged H chain, pre-treated cells with NEM at 37 °C for 15 min, and lysed them in the presence of NEM. Proteins from NEM-treated cells were subjected to TCEP treatment to reduce possible disulfide bonds and then counter-modification with maleimide-polyethylene glycol (Mal-PEG), a sulfhydryl-alkylating reagent with a MW of ~2 kDa. As shown in the schematic presentation of the reactions (Fig. 6c), NEM-unmodified proteins retaining free thiols in the H chain were PEGylated and their mobility was shifted accordingly, whereas proteins with H chains modified by NEM were resistant to counter-modification by Mal-PEG, and did not undergo a mobility shift. Thus, the extent of disulfide bond formation in the H chain can be assessed by the degree of counter-modification with Mal-PEG. Immunoblotting analysis of lysates subjected to reducing SDS-PAGE using anti-HA tag displayed a mobility shift, with bands larger than the native molecular mass (50 kDa) due to Mal-PEG labeling (as indicated by the arrow; Fig. 6d, left panel, lane 1). This demonstrates that the H chain can be oxidized to some degree in the cytosol, indicating that disulfide bonds are partially formed in the H chain of cytosolically expressed IgG. By contrast, in H chains not treated with Mal-PEG, a major band of 50 kDa was observed (Fig. 6d, left panel, lanes 2, 4, 6). As expected, compared with NEM-treated H chains (Fig. 6d, left panel, lane 1), NEM-untreated H chains were modified to a greater extent by Mal-PEG (Fig. 6d, left panel, lane 5). Furthermore, TCEP treatment to reduce possible disulfide bonds in NEM-unmodified proteins increased counter-modification by Mal-PEG, as expected (Fig. 6d, left panel, lane 1 *versus* 3). In parallel, immunoblotting analysis of total PEGylated proteins using anti-PEG antibody was performed as a control to ensure that lysates did indeed react with NEM and TCEP during each step (Fig. 6d, right panel). As expected, PEGylated proteins were decreased in NEM-treated lysates compared with NEM-untreated lysates (Fig. 6d, right panel, lane 1 *versus* 5), and increased in TCEP-treated lysates compared with TCEP-untreated lysates (Fig. 6d, right panel, lane 1 *versus* 3).

Meanwhile, the existence of free thiol groups on H chains was also confirmed by ELISA antigen-binding activity experiments (Fig. 6e). The antigen-binding activity of cytosolically expressed IgGs was dramatically reduced in NEM-treated lysates, revealing reduction by 33% for 3D8 IgG, 40% for 2C281 IgG, and 97% for 6C407 IgG. Alkylation of Cys residues of V regions makes the antibody more hydrophobic and could induce the unfolding of antigen-binding site, leading to the change the antigen binding properties of the antibody. Therefore, this significant change in antigen-binding activity of IgGs by NEM-based alkylation demonstrates the existence of free thiols in IgGs expressed in the cytosol. By contrast, the activity of ER-directed IgGs was only slightly changed or unchanged following NEM treatment, indicating that most of the thiol groups of Cys residues were oxidized.

### IgG1s expressed in the cytosol or via the ER assemble differently

**Figure 7:**
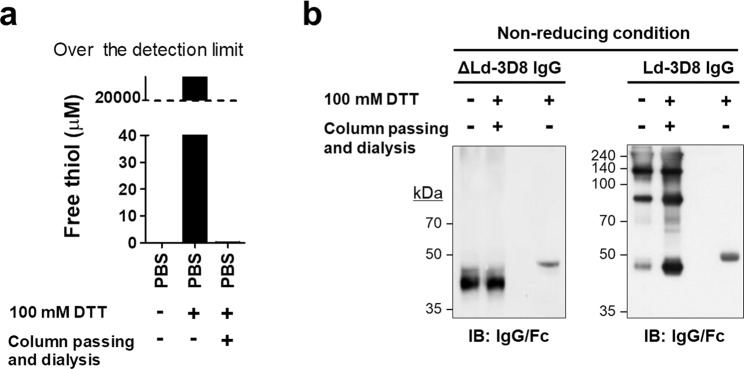
IgG1s expressed in the reducing cytosol and via the oxidizing ER assemble differently. (**a**) The concentration of free thiols in PBS was measured before and after DTT removal steps. (**b**) Immunoblotting analysis of the assembly pattern of H and L chains following DTT removal. HEK293 cells transfected with KV10ΔLd-3D8 IgG or KV10Ld-3D8 IgG. Lysates of transfectants were treated with 100 mM DTT for 30 min at room temperature, followed by DTT removal steps comprising passage through a desalting column and dialysis against PBS at 4 °C for 24 hours. Lysates were separated by non-reducing SDS-PAGE, and the H chain was detected by immunoblotting with anti-IgG/Fc antibody.

To investigate whether there are any distinct differences in the properties of IgG1s expressed in the reducing cytosol and the oxidizing environment of the ER, excluding differences due to glycosylation and disulfide bond formation, we performed a reversible redox reaction *in vitro*. Aliquots of ΔLd-3D8 IgG and Ld-3D8 IgG cell lysates were treated with 100 mM DTT for 30 min to allow full reduction, and DTT was then removed from the lysate by passing through a desalting column equilibrated with phosphate-buffered saline (PBS) followed by extensive micro-dialysis against PBS at 4 °C for 24 hours. Depletion of DTT was achieved by passing through the column, since free thiols were undetectable in the PBS control (Fig. 7a). Proteins were subjected to immunoblotting analysis in non-reducing and denaturing conditions using anti-IgG/Fc antibody (Fig. 7b). In the presence of the reducing agent DTT, only a single protein band with a MW of ~50 kDa was detected (Fig. 7b, lane 3). When DTT was omitted (Fig. 7b, lane 2), the H chains of ΔLd-3D8 IgG and Ld-3D8 IgG cell lysates returned to their original protein band patterns (Fig. 7b, lane 1). Thus, upon DTT removal from fully reduced cell lysates, the H chain of ΔLd-3D8 IgG formed only partial intrachain disulfide bonds (also shown for the fully reduced H chain, Fig. 7b, lane 3), whereas the H chains of Ld-3D8 IgG partially formed both interchain and intrachain disulfide bonds. The H chain band pattern of ΔLd-3D8 IgG was not altered from that of Ld-3D8 IgG, even though the time for spontaneous oxidation in air was more than sufficient (24 hours), and *vice versa*. Given that proteins tend to reside in their lowest energy conformation, this phenomenon indicates that the protein conformation differs between IgG1s expressed in the reducing cytosol and the oxidizing environment of the ER, despite identical primary structures.

## Discussion

**Figure 8:**
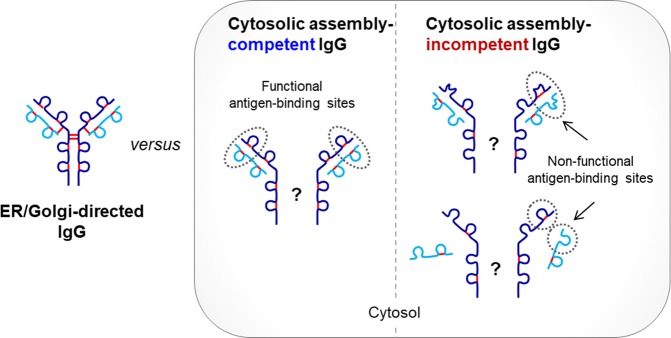
Prosed structure and functionality of full-size IgG intrabodies expressed in the cytosol of mammalian cells. IgGs expressed in the cytosol of HEK293 cells can be either assembly-competent or -incompetent, depending on the intrinsic properties of the V regions. Partial intrachain disulfide bond formation occurs in both H and L chains of cytosolic IgG intrabodies, whereas interchain disulfide bond formation between H and L chain is absent and dispensable for functional assembly. Interchain disulfide bond formation between H chains remains unknown.

Herein, we demonstrated that the reducing environment of the cytosol of mammalian cells can to some extent facilitate the correct folding of some cytosolic assembly-competent IgGs, but not a cytosolic assembly-incompetent IgG1 antibody such as a 10C358 (the results are summarized in Fig. 8). Based on a report that exposure of Fv fragments to reducing conditions *in vitro* results in irreversible denaturation of both V_H_ and V_L_^11^, V_H_ and V_L_ domains of 10C358, which is directed to the reducing cytosol, may undergo denaturation that prevents correct assembly. Until recently, predicting antibodies that are cytosolic assembly-competent was impossible based on V region sequences, and empirical research was necessary to isolate in-cell workable IgGs. Functioning antibodies expressed in the cytosol have now been reported, including Fab and IgG in the yeast *Saccharomyces cerevisiae*^23,24^, an IgG and its scFv in oocytes of the amphibian *Xenopus laevis*^25^, IgM in the green alga *Acetabularia mediterranea*^26^, scFv in plants^27–29^, full-length IgM and IgG in mammalian cells^17–19^, and scFv and single-domain antibodies in plant and animal cells^30,31^. However, it is possible that only cytosolic assembly-competent antibodies were analyzed in these studies. Our finding that V domains determine the functionality of the full-length IgG intrabody appear to be consistent with previous findings in which the sequence of V domains appears to determine the soluble expression of human scFvs and camelid V_HH_ in the mammalian cell cytoplasm^32^.

We observed that the structural integrity of the C region is required for H:L association and correct formation of the antigen-binding site (Fig. 3). This mutual effect between V and C regions on the functional assembly of cytosolic IgG was also demonstrated in a previous report which strong interactions between C_H_ and C_L_ chains induces the association between V_H_ and V_L_ domains, with a dissociation constant (from 10^−5^ M to 10^−8^ M) that is not sufficient to maintain domain association at low protein concentrations and under relatively stringent conditions^33^. Moreover, individual V_H_V_L_ and C_H_C_L_ units were shown to be mutually stabilized by a high degree of cooperation between the V_H_V_L_ and C_H_1C_L_ interfaces of Fab fragments. Some intrinsically stable scFvs can correctly fold in the reducing cytosol without formation of the usual two intrachain disulfide bonds, but others cannot^11,34–36^. Thus, functional assembly of intrabodies might be less rare among full-length IgGs than scFv forms because C regions can contribute to the assembly of V regions.

We do not know whether cytosolic IgG intrabodies were expressed as whole (2H + 2H) or half (1H + 1L) forms under our experimental conditions, but speculate that 2H + 2L forms would predominate when IgGs are expressed in the cytosol because hinge disulfide bonds are not essential for H:H association due to the pivotal role of the C_H_3 domain in the assembly of secreted human IgG1. Thus, non-covalent interactions between C_H_3 domains are sufficiently strong (*K*_*D*_ ~10^−10^ m to ~10^−14^ m) to maintain H:H association in the absence of hinge disulfide bonds, and even intrachain disulfide bonds within the C_H_3 domain are not required for C_H_3 dimerization^37–40^, although these results refer to secreted human IgG1 and its fragments. Furthermore, interchain disulfide bonds were not essential for H:L association of the cytosolic assembly-competent 3D8 IgG1 (Fig. 4), and the same is true for secreted IgGs that lack interchain disulfide bonds but remain non-covalently associated under native conditions^41^. Cys residues responsible for intrachain disulfide bond formation were partially oxidized in cytosolic 3D8 IgG (Fig. 5), suggesting that complete intrachain disulfide bond formation is not required for cytosolic IgG function. The same is also true for ER-directed IgGs, in which only some of the Cys residues capable of forming inter- and intrachain disulfides do so in C and V domains during protein folding in the ER^41–46^. The redox state has only been studied for two scFv cases; disulfide bond formation was observed in the cytosol of plant cells^29^, but not in the cytosol of *Xenopus* oocytes^47^. Our current results provide the first evidence for the complete lack of interchain disulfide bonds and partial intrachain disulfide bond formation in IgG1s expressed in the cytosol of mammalian cells.

In the case of human IgG1 secreted via the ER, interchain disulfide bonds are more susceptible to reduction than intrachain disulfide bonds, with susceptibility ordered C_H_1-C_L_ > upper C_H_2-C_H_2 > lower C_H_2-C_H_2^48^. This difference in susceptibility is believed to be due to differences in the degree of exposure of Cys residues to solvent. By contrast, little is known about the susceptibility of free sulfhydryls in cytosolically expressed IgGs to oxidation. We found no evidence of interchain disulfide bon groups promoted by ROS d formation in cytosol-directed IgG1, implying resistance to oxidation, and hence, interchain disulfide bond formation, in air (Figs. 4 and 5). This was quite unexpected because Cys residues involved in interchain disulfide bond formation are generally believed to be highly exposed to solvent and more prone to being reactive than buried Cys residues involved in intrachain disulfide bonds.

The fine structure of IgGs expressed in the cytosol may differ from IgGs within the ER. Non-native folding and assembly of cytosol-directed IgGs can be promoted by cytosolic molecular chaperons such as Hsp70 and TRiC family members during protein synthesis^49^. Meanwhile, oxidative folding and assembly are promoted by molecular chaperon Bip and protein disulfide isomerase (PDI) that catalyze the formation of disulfides (oxidase activity) through direct thiol-disulfide exchange reactions (RS–SR + R′SH ⇌ R′S–SR + RSH) and the rearrangement of incorrectly formed disulfide bonds (isomerase activity) in the ER^8^. Protein folding in the ER provides the driving force for disulfide formation and, conversely, disulfide bonds influence the thermodynamics of protein folding and maintain protein integrity^8^. Therefore, cytosol-directed IgGs may undergo their unique folding process by lowering the entropy in the absence of PDI, leading to distinct redox properties (Fig. 6).

Reducing conditions in the cytosol are achieved primarily by two semi-independent systems, the glutathione/glutaredoxin (Grx) system and the thioredoxin (Trx) system, which regulate the glutathione/glutathione-disulfide ratio (GSH:GSSG) and the reduced/oxidized Trx ratio, respectively^50^. Reducing components such as GSH and Trx keep disulfide bonds reduced, resulting in a low thiol-disulfide redox potential and less chance of forming stable disulfide bonds. The redox balance can be altered transiently by naturally-occurring metabolic compounds such as reactive oxygen species (ROS)^2,7^, which can result in the formation of disulfide bonds in cytosolic proteins in a non-enzymatic manner. Non-enzymatic disulfide bonds in proteins can be formed *in vitro* and *in vivo* following oxidation of sulfhydryl (–SH) groups promoted by ROS (2 RSH ⇌ RS–SR + 2H^+^ + 2 e^−^) following the action of cysteine sulfenic acid (Cys-SOH), a reaction intermediate spontaneously generated from cysteine thiols^7,22^. We showed that partial intrachain disulfide bond formation in H and L chains of IgGs can occur in the cytosol of human cells. These results raise the question of whether the formation of intrachain disulfide bonds is due to a certain GSH:GSSG ratio and/or reduced/oxidized thioredoxin ratio, set by the equilibrium state of the cytosol, when conditions are not sufficiently reducing to prevent disulfide bond formation. Otherwise, once folded into the native IgG structure, disulfide bonds formed transiently in the presence of ROS might become inaccessible to the reducing systems in the cytosol. Indeed, non-enzymatic formation of intrachain disulfide bonds in cytosolically expressed IgGs can lead to non-specific and irreversible impairment of protein structure and function, and this may influence the functionality of IgG antibodies to a certain extent.

## Methods

### Plasmid construction

Genes encoding V regions of mouse monoclonal antibodies (3D8, 2C281, 6C407 and 10C358) were cloned into the KV10 plasmid for expression of chimeric IgG1s. The complete cDNA sequences of V regions of these antibodies have been deposited in GenBank (3D8 V_H_, GenBank accession number AAF79128; 3D8 V_L_, AAF79129; 2C281 V_H_, MH638364; 2C281 V_L_, MH638365; 6C407 V_H_, MH638366; 6C407 V_L_, MH638367; 10C358 V_H_, MH638368; 10C358 V_L_, MH638369; pseudo Vκ, MH638370). Alignments of their V region sequences are shown in Supplementary Fig. S1.

The KV10 vector contains human Cγ1, Cγ2, Cγ3, and Cκ genes under the control of two individual CMV promoters (P_CMV_) that allow simultaneous expression of H and L chains. The KV10 plasmid was designed as a cassette vector to permit cloning of all types of heavy (H) and light (L) Ig chains, with or without leader sequences upstream, using specific restriction enzymes, and to facilitate individual cloning of antibody fragment gene cassettes (V_H_, V_L_, C_H_1-3 and C_L_) into the specific cloning sites. V_H_ and C_H_1-3 H chain genes were flanked with *Mfe*I/*Nhe*I and *Nhe*I/*BamH*I restriction enzyme sites, respectively, while V_L_ and C_L_ genes were flanked with *Bgl*II/*BsiW*I and *BsiW*I/*EcoR*I. For secretory expression of IgG1, KV10Ld-HL plasmids with leader sequences were constructed. V_H_ genes with the V_H_3 gene family leader sequence were cloned upstream of C_H_1 using *Mlu*I/*Nhe*I restriction sites. V_L_ genes with the leader sequence from the V_κ_1 gene family were cloned upstream of the respective C_L_ using *Dra*III/*BsiW*I restriction sites. For cytoplasmic expression of IgG1, leader-deficient KV10ΔLd-HL plasmids were constructed. V_H_ genes were cloned upstream of C_H_1 using *Mfe*I/*Nhe*I restriction sites, and V_L_ genes were cloned upstream of C_L_ using *Bgl*II/*BsiW*I restriction sites.

Plasmids for expression of 3D8 IgG variants were constructed as follows; ΔLd-*HL was constructed from the KV10ΔLd-HL vector to express 3D8 Ig protein lacking disulfide bonds between the two H chains by replacing Cys11 and Cys14 of the H chain hinge region (EPKSCDKTHT**C**PP**C**P) with Ser in the ΔLd-HL vector. ΔLd-*H*L was constructed from the ΔLd-*HL vector to express modified H and L chains incapable of forming interchain disulfide bonds (both H-H and H-L disulfide bonds) by replacing Cys107 of Cκ with Ser. ΔLd-H*L expressing WT H chain and modified L chain was constructed by cloning the modified L chain, in which Cys107 of Cκ was replaced with Ser, into ΔLd-HL. ΔLd-H and ΔLd-L that express H or L alone, respectively, were constructed by inserting two subsequent stop codons at the 5′ end of V_H_ and V_L_ genes. To express 3D8 IgGs with HA-tagged H chains and Flag-tagged L chains in the cytosol of HEK293 cells, we constructed ΔLd-3D8 IgG/H-HA and ΔLd-3D8 IgG/L-Flag by replacing the H chain gene with the synthesized H-HA chain gene using *Nhe*I and *BamH*I restriction sites, and replacing the L chain gene with L-Flag chain gene using *BsiW*I and *EcoR*I restriction sites.

To express 3D8 IgG1 which is unable to form intrachain disulfide bonds, we constructed Ld-*^#^H*^#^L and ΔLd-*^#^H*^#^L plasmids encoding H and L chains in which all 16 Cys residues, responsible for both inter- and intrachain disulfide bond formation, were mutated to Ser. For construction of ΔLd-*^#^H*^#^L, genes encoding V_H_-C_H_1-C_H_2-C_H_3 and V_L_-C_L_ variants were synthesized and cloned into the KV10 vector using *Mfe*I/*BamH*I restriction sites for the H chain, and *Bgl*II/*EcoR*I restriction sites for the L chain. For construction of Ld-*^#^H*^#^L, genes encoding the leader-V_H_ variant and leader-V_L_ were constructed by cloning the synthesized H and L genes into *Mfe*I/*Nhe*I and *Bgl*II/*BsiW*I restriction sites, respectively.

To construct plasmids for ΔLd-IgG-Cw variants in which C_H_ and C_L_ chains are unable to form any disulfide bonds, 12 Cys residues in C_H_ and C_L_ chains, responsible for both inter- and intrachain disulfide bond formation, were mutated to Ser. The C_H_1-C_H_2-C_H_3 and C_L_ genes of ΔLd-HL were replaced with the C_H_1-C_H_2-C_H_3 variant gene from ΔLd-*^#^H*^#^L using *Nhe*I/*BamH*I and *BsiW*I/*EcoR*I restriction sites, respectively. Plasmids for expression of IgGs composed only of C regions without V regions, designated Ig-Cw plasmids, were constructed by cloning the synthesized WT and variant C_H_1-C_H_2-C_H_3 and C_L_ chain genes into the ΔLd-HL vector using *Mfe*I/*BamH*I restriction sites for the H chain and *Bgl*II/*EcoR*I restriction sites for the L chain.

### Preparation of cell lysates

HEK293 (ATCC; cat# CRL-1573) cells, obtained from the American Type Culture Collection (ATCC), were seeded in 100 mm dishes at a density of 5 × 10^6^ cells/dish 24 hours prior to transfection with a plasmid. Plasmid DNA (20 μg) was pre-incubated with polyethylenimine (PEI) reagent (96 μg) at room temperature for 10 min, then inoculated into cells in 10 ml of Opti-MEM (Gibco; cat# 31985-070) at 37 °C for 24 hours. At 24 hours after transfection, cells were treated with 10 μM MG132 for 12 hours or 50 mM N-ethylmaleimide (NEM) reagent (ThermoFisher; cat# 23030) for 15 min if necessary. After three washes with cold PBS, transfected cells in cold PBS (0.6 ml/dish) were harvested using a scraper. Cells were collected by centrifugation at 850 × g at 4 °C for 5 min, and lysed by sonication at 30% amplitude using three 15 s pulses (Epishear) in ice-cold PBS containing protease inhibitor cocktail (Roche; cat# 11697498001). Where necessary, cells were lysed in PBS supplemented with protease inhibitor cocktail plus 100 mM NEM. Supernatants were obtained by centrifugation at 16,000 × g at 4 °C for 10 min. Protein concentration in cell lysates was measured using a BCA Protein assay kit (ThermoFisher; cat# 23227).

### Immunoprecipitation

Aliquots (500 μg) of cell lysate, prepared as described above in ‘*Preparation of cell lysates*’, was subjected to IP with Protein A/G coupled to resin (ThermoFisher; cat# 26146) according to the manufacturer’s instructions. After washing the resin, immunoprecipitated proteins were eluted and resolved by SDS-PAGE, followed by immunoblotting.

### Immunoblot analysis

For SDS-PAGE analysis under non-reducing conditions, samples were diluted 1:1 in 2× Laemmli sample buffer (Bio-Rad). For reducing conditions, samples were diluted 1:1 in 2× Laemmli sample buffer supplemented with 2.5% 2-mercaptethanol or 100 mM DTT. In both cases, samples were heated at 100 °C for 10 min, then loaded on 8%, 10%, or 4–20% acrylamide gels. Following electrophoresis, resolved proteins were transferred onto polyvinylidene fluoride membranes (Millipore). Membranes were rinsed with PBS, then blocked with 5% milk (w/v) in TRIS-buffered saline (TBS, 50 mM TRIS-Cl, 50 mM NaCl, pH 7.2) containing 0.05% Tween-20 (TBST) at 4 °C overnight. Membranes containing Ig proteins were probed with primary goat anti-human IgG/Fc antibody (Pierce; cat# 31125) plus rabbit anti-human Ig κ chain antibody (Abcam; cat# ab134083) in 5% milk-TBST at 4 °C for 12 hours. After washing three times with PBS containing 0.05% Tween-20 (PBST), membranes were incubated with secondary horseradish peroxidase (HRP)-conjugated anti-goat IgG (Invitrogen; cat# 81-1620) plus HRP-conjugated anti-rabbit IgG (Invitrogen; cat# 81-6120) for 1 h. After washing five times with TBST, protein signals on membranes were visualized with an ECL kit (GE Healthcare; cat# RPN2106). Where necessary, membranes were probed with primary mouse anti-HA antibody (Millipore; cat# 05-904) plus mouse anti-Flag antibody (Sigma-Aldrich; cat# F3165), followed by HRP-conjugated horse anti-mouse IgG (Cell Signaling; cat# 7076).

### ELISA

Lysates of transfected cells were prepared as described above in ‘*Preparation of cell lysates*’. To assess the association of Ig H and L chains in cells, three different ELISA protocols were used. In the first method, wells of a 96-well polystyrene plate were coated with 100 μl (2 μg/ml) of goat anti-human IgG/Fc antibody (Abcam; cat# ab97221) for 1 hour at room temperature, washed three times with TBST, and blocked with 3% BSA for 1 hour at room temperature. Wells were subsequently incubated with lysates of transfected cells (100 μl) for 1 hour at room temperature, rabbit anti-human Cκ antibody (Abcam; cat# ab125919), and alkaline phosphatase (AP)-conjugated goat anti-rabbit IgG/Fc-specific antibody (Pierce; cat# 31341). Each incubation step was followed by washing three times with TBST. Otherwise, the plate coated with 2 μg/ml of rabbit anti-human Cκ antibody or Protein L (BioVision; cat# 6530) was incubated with lysates of transfected cells, followed by AP-conjugated goat anti-human IgG/Fc-specific antibody (Sigma-Aldrich; cat# A9544). Finally, p-nitrophenyl phosphate (Sigma-Aldrich; cat# N2765) solution (1 mg/ml in 0.1 M glycine, 1 mM ZnCl_2_ and 1 mM MgCl_2_, pH 10.3) was added to each well and the absorbance at 405 nm was measured using a microplate reader (Molecular Devices). Polyclonal human IgG (Sigma-Aldrich; cat# I8640) was used as a positive control. Where necessary, lysates were heated for 10 min at 100 °C.

To assess the formation of an intact antigen-binding site in 3D8 IgG1, lysates of transfected cells were incubated in a 96-well plate coated with 1 μg/ml calf thymus DNA (Sigma-Aldrich; cat# 89380). Otherwise, lysates of transfected cells were incubated in a plate coated with O2F3 IgM (10 μg/ml) specific for the conformational idiotype of the 3D8 antibody, and subsequently treated with rabbit anti-human Cκ. Bound 3D8 IgG1 was then detected using AP-conjugated goat anti-human IgG/Fc antibody (Sigma-Aldrich; cat# A9544).

To assess the antigen-binding activity of anti-KIFC1 antibodies (2C281, 6C407 and 10C358), lysates of transfected HEK293 cells were incubated in a 96-well plate coated with synthetic peptide antigens (1 μg/ml). Synthetic peptide antigens for 2C281 consisted of amino acids 70–81 (PSLTTVPQTQGQ) of KIFC1. Peptide antigens for 6C407 and 10C358 consisted of amino acids 43–54 (EDGLEPEKKRTR) and 99–110 (IATGLKNQKPVP) of KIFC1 (summarized in Supplementary Fig. S2). Bound anti-KIFC1 antibodies were detected with AP-conjugated goat anti-human IgG/Fc antibody (Sigma-Aldrich; cat# A9544). Where necessary, lysates of transfected HEK293 cells were prepared in the presence of 100 mM NEM.

### Confocal microscopy

To analyze colocalization of cytosolically expressed 3D8 IgG and O2F3 antibodies (mouse IgM specific for conformational V regions of 3D8)^20^, HEK293 cells were seeded on poly L lysine-coated glass coverslips in 24-well plates at a density of 4 × 10^4^ cells/well, prior to transfection with 8 μg KV10ΔLd-3D8 plasmid using 2 μl Lipofectamine 2000 reagent (Life Technologies). At 24 hours after transfection, cells were washed three times with ice-cold PBS, pH 7.2) and fixed with 4% paraformaldehyde in PBS for 10 min at 4 °C. After washing cells three times with PBS, cell membranes were permeabilized with P buffer consisting of 1% bovine serum albumin (BSA), 0.1% saponin, and 0.1% sodium azide in PBS for 10 min at room temperature. Cells were incubated overnight at 4 °C with O2F3 antibody, and then with an Alexa Fluor 647-conjugated rat anti-mouse IgM/µ chain-specific antibody (BioLegend; cat# 406526). After each incubation for 1 hour at 4 °C, cells were washed three times with ice-cold PBS. Finally, cell nuclei were stained with Hoechst 33342 (Vector Laboratories) for 30 min at room temperature. Thereafter, cells on coverslips were mounted with Vectashield mounting medium (Vector Laboratories). Images were obtained using a laser scanning confocal fluorescence microscope (LSM710, Carl Zeiss).

For analysis of colocalization of anti-KIFC1 IgG intrabodies and cellular KIFC1 molecules, HeLa (ATCC; cat# CCL-2) cells stably expressing GFP-tagged KIFC1 were seeded onto glass cover slips and transfected with plasmid encoding KV10ΔLd-2C281, KV10ΔLd-6C407, or KV10ΔLd-10C358. At 24 hours after transfection, cell synchronization was performed to enrich cells in mitosis. To arrest the majority of cells in G1/S, cells were treated with 2 mM thymidine for 18 hours, washed with PBS three times, and placed in fresh medium for 7 hours. To arrest cells at the G2/M phase border, cells were incubated with 9 μM CDK inhibitor RO3306 (Sigma-Aldrich; cat# SML0569) for 2 hours, and then placed in fresh medium for 30 min, fixed, and permeabilized. Cells were incubated with goat anti-human IgG/Fc-specific antibody (Abcam cat# ab97221) followed by tetramethylrhodamine isothiocyanate (TRITC)-conjugated rabbit anti-goat IgG.

### Purification of Ig proteins

O2F3 (mouse IgM) was purified from the culture supernatant of O2F3 hybridoma cells cultured in RPMI 1640 media supplemented with 10% FBS for 7 days at 5% CO_2_ and 37 °C. O2F3 IgM protein was purified from the culture supernatant by affinity chromatography using agarose-goat anti-mouse IgM/μ chain-specific resin (Sigma-Aldrich; cat# A4540) according to the manufacturer’s instructions.

ER-directed chimeric 3D8 IgG1 protein was purified from the culture supernatant of FreeStyle 293-F cells (ThermoFisher; cat# 12338). FreeStyle 293-F cells (100 ml) at a cell density of 1 × 10^6^ cells/ml were seeded 24 hours prior to transfection in a 500 ml flask (Corning; cat# 431145) to ensure they reached the appropriate cell density (2 × 10^6^ cells/ml) at the time of transfection. Culturing was performed in serum-free FreeStyle 293 medium (Invitrogen; cat# 12338) at 8% CO_2_ and 37 °C with shaking at 130 rpm. Plasmid (200 μg) encoding a pair of Ig H and L chains was transiently introduced into the 100 ml FreeStyle 293-F cell culture (2 × 10^6^ cells/ml) with PEI reagent (25 kDa, Polyscience; cat# 23966-2). Specifically, PEI reagent (400 μg) was incubated with plasmid DNA (200 μg) at room temperature for 10 min, and mixtures were inoculated into 100 ml of cells to achieve a 4 μg/ml final PEI concentration. After 6–7 days, the culture supernatant was harvested by centrifugation, and Ig proteins were purified by affinity chromatography using agarose-Protein A (GE Healthcare; cat# 17-1279-02) according to the manufacturer’s instructions.

### Analysis of the redox state of IgGs

HEK293 cells, seeded in 100 mm dishes at a density of 5 × 10^6^ cells/dish, were transfected by incubating with a mixture of plasmid DNA (20 μg) plus PEI reagent (96 μg) in 10 ml Opti-MEM at 37 °C for 24 hours. Cells were pre-incubated with NEM at a final concentration of 50 mM at 37 °C for 15 min and lysed by sonication as described above in ‘*Preparation of cell lysates*’ in the presence of 100 mM NEM. After incubation of lysates at 37 °C for 30 min, supernatants were obtained by centrifugation at 16,000 × g at 4 °C for 10 min. Aliquots of supernatants were passed through a desalting column (ThermoFisher; cat# 89882) equilibrated with PBS to remove free NEM. After treatment of supernatants with TRIS-(2-carboxyethyl) phosphine (TCEP) at a final concentration of 5 mM at 37 °C for 1 hour, samples were incubated with 10 mM maleimide conjugated to polyethylene glycol (PEG) with a MW of 2 kDa at 37 °C for 1 hour. Samples were diluted 1:1 in 2× Laemmli sample buffer supplemented with 2.5% 2-mercaptethanol, heated at 100 °C for 10 min, and then loaded onto acrylamide gels. Samples from the ΔLd-3D8 IgG/H-HA lysate were loaded onto 8% SDS-acrylamide reducing gels. Immunoblotting analysis of the H chain was performed with mouse anti-HA antibody (Millipore; cat# 05-904) plus HRP-conjugated anti-mouse IgG antibody (Cell Signaling; cat# 7076). Immunoblotting analysis of PEG was carried out with rabbit anti-PEG (Abcam; cat# ab51257) plus HRP-conjugated anti-rabbit IgG antibody (Zymed; cat# 81-6120).

### Analysis of assembly dynamics

HEK293 cells were transfected with KV10ΔLd-3D8 IgG or KV10Ld-IgG at 37 °C for 24 hours. Aliquots of transfectant lysates were treated with 100 mM DTT for 30 min, then passed through a desalting column (ThermoFisher; cat# 89882) equilibrated with PBS, followed by micro-dialysis against PBS using a micro-dialyzer (ThermoFisher; cat# 88260) at 4 °C for 24 hours. Proteins were subjected to immunoblotting analysis in non-reducing and denaturing conditions using anti-IgG/Fc antibody. To validate the complete removal of DTT in the above column and dialysis steps, 100 mM DTT in PBS was passed through the desalting column then subjected to dialysis against PBS, and the concentration of DTT was determined using a free thiol detection kit (Abcam; cat# ab112158).

## Supplementary information

Supplementary Information.
