# Supplementary material for: Assembly and Folding Properties of Cytosolic IgG Intrabodies
Source: Sci Rep. 2020 Feb 7;10:2140. doi: 10.1038/s41598-020-58798-7 (PMC7005851; doi:10.1038/s41598-020-58798-7)
Supplement: Supplementary file 1 — Supplementary Information. [file 41598_2020_58798_MOESM1_ESM.docx]

**Supporting information**

**Assembly and Folding Properties of Cytosolic IgG Intrabodies**

Youngsil Seo^1,2^ *, Yeonjin Lee^1^ *, Minjae Kim^1^, Hyunjoon Park^1^, and Myung-Hee Kwon^1,2 †^

^1^Dept. of Biomedical Sciences, Graduate School, Ajou University, 206 World cup-ro, Yeongtong-gu, Suwon 16499, Gyeonggi-do, South Korea.

^2^Dept. of Microbiology, Ajou University School of Medicine, 206 World cup-ro, Yeongtong-gu, Suwon 16499, Gyeonggi-do, South Korea.

*These authors contributed equally to this work.

^†^Correspondence: Department of Microbiology, Ajou University School of Medicine, 206 World Cup-ro, Yeongtong-gu, Suwon 16499, South Korea. Tel: +82-31-219-5074; Fax: +82-31-219-5079; E-mail: [kwonmh@ajou.ac.kr](mailto:kwonmh@ajou.ac.kr)

**Supporting information contains:**

Supplementary Figure 1 (Fig. S1) and legend

Supplementary Figure 2 (Fig. S2) and legend

Supplementary Figure 3 (Fig. S3): Raw western blot of Figure 1d

Supplementary Figure 4 (Fig. S4): Raw western blot of Figure 3b

Supplementary Figure 5 (Fig. S5): Raw western blot of Figure 4f

Supplementary Figure 6 (Fig. S6): Raw western blot of Figure 5f

Supplementary Figure 7 (Fig. S7): Raw western blot of Figure 5g

**Fig. S1**

**
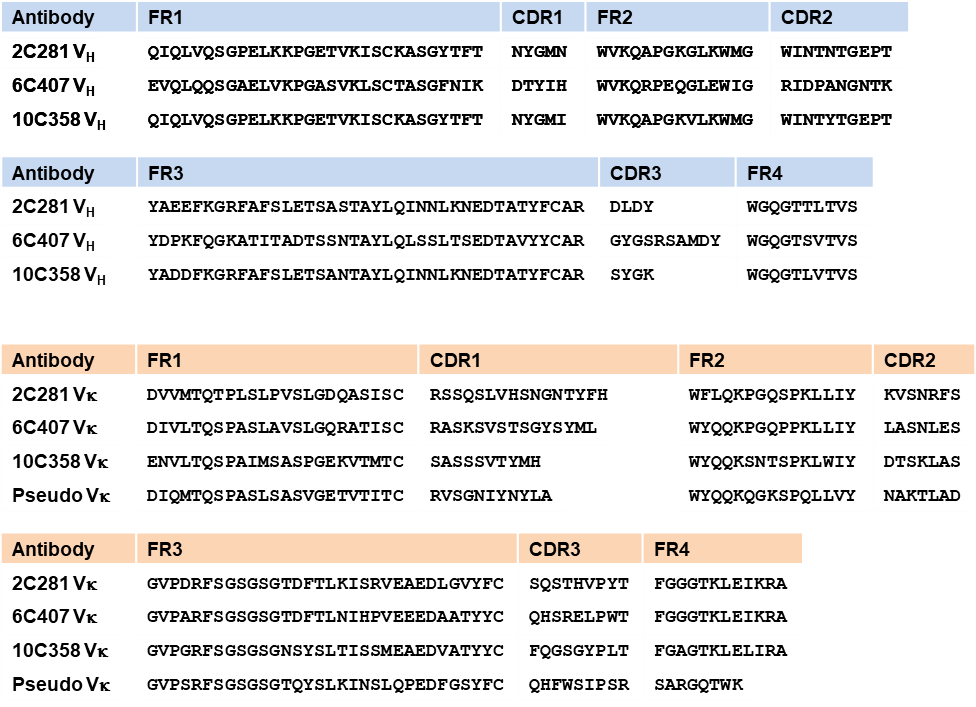
**

**Supplementary Fig. S1. Amino acid sequence alignments of variable domains (V_H_ and V_L_).** Amino acid sequences of variable regions for three mouse anti-KIFC1 antibodies (2C281, 6C407, and 10C358) and a pseudo mouse sequence are aligned. The positions of complementarity determining regions (CDRs) and framework regions (FRs) are indicated according to the ImMunoGeneTics (IMGT) numbering system.

**Fig. S2**

**
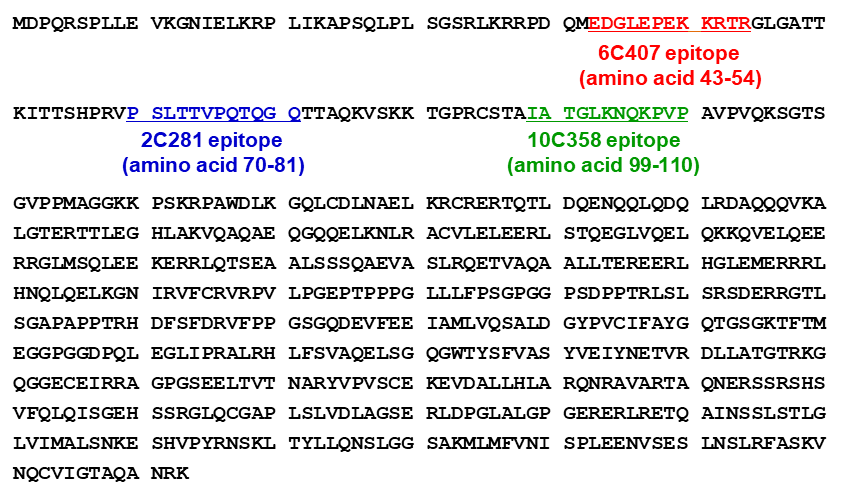
**

**Fig. S2. Amino acid sequences of the KIFC1 protein and epitopes recognized by antibodies.** The epitope sequences recognized by 6C407, 2C281, and 10C358 antibodies are indicated by red, blue, and green amino acids, respectively.

**Fig. S3. Raw western blot of Figure 1d**

**
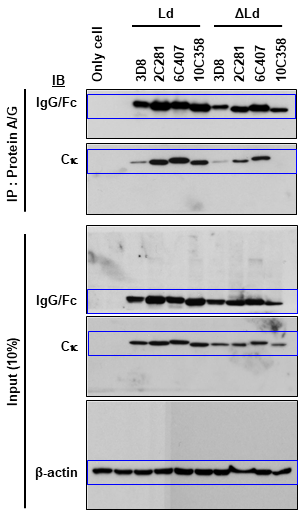
**

**Fig. S4. Raw western blot of Figure 3b**

**
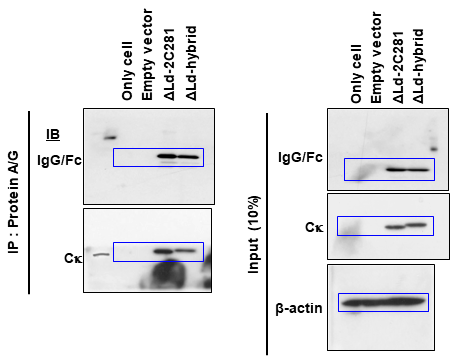
**

**Fig. S5. Raw western blot of Figure 4f**

**
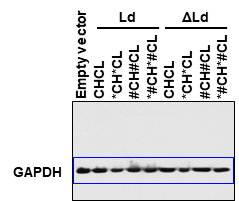
**

**Fig. S6. Raw western blot of Figure 5f**


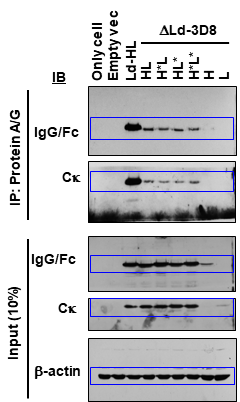


**Fig. S7. Raw western blot of Figure 5g**

**
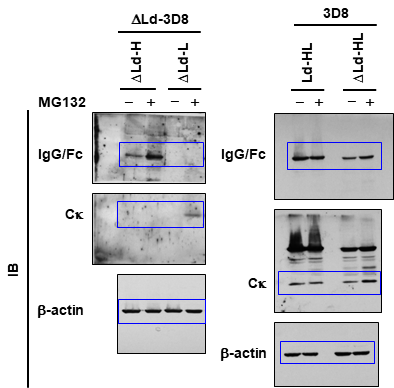
**
